# Effectiveness of ginger in reducing metabolic levels in people with diabetes: a randomized clinical trial*

**DOI:** 10.1590/1518-8345.3870.3369

**Published:** 2020-10-09

**Authors:** Gerdane Celene Nunes Carvalho, José Claudio Garcia Lira-Neto, Márcio Flávio Moura de Araújo, Roberto Wagner Júnior Freire de Freitas, Maria Lúcia Zanetti, Marta Maria Coelho Damasceno

**Affiliations:** 1 Universidade Estadual do Piauí, Departamento de Enfermagem, Picos, PI, Brazil.; 2 Universidade Federal do Ceará, Departamento de Enfermagem, Fortaleza, CE, Brazil.; 3 Scholarship holder at the Coordenação de Aperfeiçoamento de Pessoal de Nível Superior (CAPES), Brazil.; 4 Fundação Oswaldo Cruz, Saúde Pública, Eusébio, CE, Brazil.; 5 Universidade de São Paulo, Escola de Enfermagem de Ribeirão Preto, PAHO/WHO Collaborating Centre for Nursing Research Development, Ribeirão Preto, SP, Brazil.

**Keywords:** Ginger, Type 2 Diabetes Mellitus, Type 2, Blood Glucose, Lipids, Clinical Trial, Placebo, Gengibre, Diabetes Mellitus, Tipo 2, Glicemia, Lipídeos, Ensaio Clínico, Placebo, Jengibre, Diabetes Mellitus, Tipo 2, Glucemia, Lípidos, Ensayo Clínico, Placebo

## Abstract

**Objective:**

to evaluate the effectiveness of ginge (Zingiber officinale) in reducing blood sugar and lipid levels in people with type 2 diabetes.

**Method:**

a randomized and double-blind clinical trial conducted with people with type 2 diabetes in primary care facilities. The study included individuals aged between 20 and 80 years old, using oral antidiabetic drugs and with HbA1c levels between 6.0% and 10%. The participants were paired 1:1, allocated in two distinct groups, and randomized in blocks, based on their HbA1c levels. In the experimental group, the participants used 1.2g of ginger and, in the control group, 1.2g of placebo, daily for 90 days. The primary outcome was a reduction in fasting blood sugar and HbA1c, and the secondary outcome was a reduction in lipids and HOMA-IR. 103 individuals completed the study, 47 in the experimental group and 56 in the control group.

**Results:**

the participants in the experimental group showed a greater reduction in the blood glucose and total cholesterol values compared to the control group.

**Conclusion:**

the use of ginger can help in the treatment of people with diabetes, and data support the inclusion of this herbal drug in the clinical practice of nurses. RBR-2rt2wy

## Abstract (pt)

**Objetivo:**

avaliar a efetividade do gengibre (*Zingiber officinale*) na redução de níveis glicêmicos e lipídicos de pessoas com diabetes tipo 2.

**Método:**

ensaio clínico randomizado, duplo cego, conduzido com pessoas com diabetes tipo 2, em unidades de atenção primária à saúde. Foram incluídos no estudo indivíduos com idade entre 20 e 80 anos, em uso de antidiabéticos orais e com valores de HbA1c entre 6,0% e 10%. Os participantes foram pareados de 1:1, alocados em dois grupos distintos e randomizados em blocos, com base nos valores de HbA1c. No grupo experimental os participantes usaram 1,2g de gengibre, e no grupo controle 1,2g de placebo, diariamente, durante 90 dias. Os desfechos primários foram a redução da glicemia venosa de jejum e HbA1c, e os secundários a redução dos lipídicos e HOMA-IR. 103 pessoas concluíram o estudo, encontrando-se 47 no grupo experimental e 56 no grupo controle.

**Resultados:**

os participantes do grupo experimental apresentaram melhor redução dos valores de glicemia e colesterol total, em comparação com o grupo controle.

**Conclusão:**

o uso do gengibre pode auxiliar o tratamento das pessoas com a diabetes, e os dados dão suporte para a inserção desse fitoterápico na prática clínica dos enfermeiros. RBR-2rt2wy

## Abstract (es)

**Objetivo:**

evaluar la eficacia del jengibre *(Zingiber officinale)* en la reducción de los niveles glucémicos y de lípidos en personas con diabetes tipo 2.

**Método:**

ensayo clínico aleatorizado y doble ciego, realizado con personas con diabetes tipo 2 en unidades de atención primaria de salud. Se incluyeron en el estudio individuos con edades comprendidas entre 20 y 80 años, que utilizaban antidiabéticos orales y con valores de HbA1c entre 6,0% y 10%. Los participantes fueron comparados de forma equitativa (1:1), asignados a dos grupos distintos y aleatorizados en bloques, basados en sus valores de HbA1c. En el grupo experimental, los participantes utilizaron 1,2 g de jengibre, y en el grupo de control 1,2 g de placebo, diariamente durante 90 días. Los resultados primarios fueron la reducción de glucemia venosa en ayunas y de HbA1c, y los resultados secundarios fueron la reducción de lípidos y del índice HOMA-IR. El estudio contó con la participación de 103 personas, 47 en el grupo experimental y 56 en el grupo de control.

**Resultados:**

los participantes del grupo experimental presentaron una mayor reducción en los valores de glucosa y colesterol total, en comparación con el grupo de control

**Conclusión:**

el uso del jengibre puede ayudar en el tratamiento de personas con diabetes, y los datos respaldan la introducción de este fitoterapéutico en la práctica clínica de los enfermeros. RBR-2rt2wy

## Introduction

The control of type 2 Diabetes Mellitus (T2DM) has been one of the main challenges for health professionals, researchers, and people with the disease^(^ 1 ^)^. Factors such as clinical inertia and lack of adherence to the prescribed therapeutic regimen appear as strong obstacles in the treatment of the disease, leading to important metabolic dysregulation^(^ 2 ^-^ 4 ^)^.

As a result, the worldwide interest in research involving the use of alternative and complementary practices has been increasing. This interest is due to factors such as the search for affinities for the use of natural products; the high price of private medical assistance, together with the high cost of the medications; precarious public assistance; and the attempt to mitigate complications related to chronic diseases, such as T2DM^(^ 5 ^-^ 9 ^)^. In this sense, ginger appears as a promising adjuvant for the treatment of T2DM, mainly acting in the regulation of lipid metabolism, in the improvement of anti-inflammatory activities, and in the modulation of insulin release and response, with minimal adverse events and increasingly effective results^(^ 10 ^-^ 13 ^)^.

Given the above, it was established as a hypothesis that the use of ginger is effective in decreasing glycemic and lipid biomarkers in people with T2DM, compared to a placebo. However, studies analyzing the effect of ginger in the treatment of people with T2DM are still scarce and so far no publications have been found on the subject in Brazil, indicating the need for more evidence to legitimize and subsidize the inclusion of this product in the clinical practice of health professionals, mainly in Primary Health Care, as a way to facilitate the control of T2DM^(^ 12 ^-^ 13 ^)^. This study aimed at assessing the effectiveness of ginger (Zingiber officinale) in reducing blood sugar and lipid levels in people with T2DM.

## Method

A randomized, double-blind, placebo-controlled, and parallel-group clinical trial (1:1) conducted from December 2017 to June 2018 in Primary Health Care Units (PHCUs) in Picos, in the Vale do Rio Guaribas region, state of Piauí, Brazil, with people diagnosed with T2DM. The PHCUs were chosen at random, by means of a draw. Units that were operating at least in the morning and afternoon shifts and that had people registered and followed-up with T2DM diagnoses participated in the draw.

The study included people diagnosed with T2DM for at least two years, aged between 20 and 80 years old, with preserved cognitive functions – according to the Mini Mental State Examination (MMSE)^(^ 14 ^)^, and undergoing treatment with oral antidiabetic drugs and glycated hemoglobin (HbA1c) between 6.0% and 10.0% in the baseline. The cut-off point established for HbA1c is justified since, with levels below 6.0%, people with T2DM already have good control of this biomarker; and, above 10.0%, these patients would already have important dysregulation, making the research unfeasible^(^ 15 ^)^.

In turn, the exclusion criteria used were the following: people using alcohol or tobacco, using any natural product to control diabetes, on insulin therapy, with the presence of chronic changes (cardiovascular, liver, kidney, gastric, or mental disorders diagnosed), and pregnant or lactating women. Chronic changes and mental disorders were assessed through information provided by the participants themselves, during assessment of the eligibility criteria, and confirmed with the health professionals in the PHCU where they were monitored. The individuals could be discontinued from the study if they experienced any adverse events. All the data was previously checked during the nursing appointments.

The study had as primary outcome the reduction in blood sugar levels (fasting glucose and HbA1c) of people with T2DM and, as secondary outcomes, the reduction in lipid levels (total cholesterol, triglycerides, LDL-cholesterol, and HDL-cholesterol) and the variation of the HOMA-IR index. In Picos there were no records on the number of people with T2DM and HbA1c levels between 6.0% and 10.0%, monitored in the PHCUs. Therefore, the sample was calculated using the mean difference between two groups using the G*Power 3.1.9.2 software, in which a significance level of 5% and a test power of 80% were set, based on a previous study^(^ 16 ^-^ 17 ^)^, which resulted in the need to include 102 individuals. However, considering potential losses, a percentage of 40% was added.

Recruitment took place between December 2017 and February 2018, in seven PHCUs in the urban area of the city. In total, 229 people were recruited and, of these, 85 were excluded after applying the eligibility criteria (24 did not have HbA1c between 6.0% and 10.0%; 15 used alcohol; 11 did not use oral antidiabetic drugs; 10 used tobacco; nine used insulin; nine had severe cardiovascular problems; and seven had some kidney disorder).

For recruitment, a team previously trained by the lead researcher held meetings with nurses, physicians, nursing technicians, and community health agents to explain the objective of the study. Invitation letters and the Free and Informed Consent Form (FICF) were handed in to the community health agents so that they could distribute them to potential research participants, giving them time to read and elaborate questions about the research. Guidance was given to people so that the FICF would only be signed after the researcher read it, and when people were sure that they would like to participate in the study.

The randomization sequence was created using a computer software and stratified by PHCU, with a 1:1 allocation in parallel groups, using random block sizes of six individuals, based on their HbA1c levels. Thus, each person was appointed to participate in a group based on chance, with an equal chance of being allocated to one of the comparison groups.

The allocation sequence was carried out by two members of the research group who did not directly participate in data collection. These researchers were responsible for randomizing the participants into blocks, preparing the bottles and numbering them. Thus, the lead researcher and the participants were blinded during the intervention. However, the statistician was fully aware of the allocation and identification of the participants. For randomization, a numerical list was generated, where the sequence of even numbers corresponded to the Experimental Group (EG), and the sequence of odd numbers, to the Control Group (CG). The group that each person was a part of was only revealed to the lead researcher after data analysis.

At the EG, each participant received a bottle containing 60 capsules of ginger (Zingiber officinale) per month, for three months. Each capsule contained 600mg of powdered ginger. The CG participants received a bottle containing 60 placebo capsules (microcrystalline cellulose) per month, for three months. Each capsule contained 600 mg of powdered microcrystalline cellulose. Both the EG and the CG were instructed to take two capsules a day, one 30 minutes before breakfast and the other 30 minutes before lunch. All the participants were instructed to use the respective products for 90 days. Both ginger and placebo capsules and bottles were identical (in order to mitigate the contamination of the participants), and contained a label with information about the dosage, expiration date of the product (greater than the intervention period), and the date of the follow-up appointment. Each bottle was numbered to facilitate the randomization process of the participants. A new bottle containing ginger or placebo capsules was delivered at intervals of 25 to 29 days. Telephone calls were made to remind the participants to fetch a new bottle from the PHCU where they were monitored.

Powdered rhizome was used to encapsulate the ginger, and the final product was 0.1% dried ginger extract. Water was used as a solvent and starch as an excipient in the extraction to obtain the raw material. Drying was done by spray dryer. As for the physical aspects, the concentration of the extract in water was 33.51%, and alcohol, 0.89%. The dosage was 0.36% for total gingerols (6-gingerol, 10-gingerol, 6-shogaol). In addition to the physical-chemical test carried out by the manufacturer, a microbiological test was carried out, showing a normal amount of bacteria, fungi and yeasts, and the purity test measured heavy metals, such as lead, copper, and antimony.

The use of ginger is authorized in Brazil and does not require authorization to be used in research studies. Therefore, the concept of “Access to the Genetic Heritage” available in Provisional Measure No. 2186-16/ 2001 does not apply. It is worth remembering that this spice, despite being originally from the island of Java, India, and China, is widely used in tropical regions of the world.

After the purchase of 0.1% dried ginger powder (Gemini Indústria de Insumos Farmacêuticos Ltda.), the weighing, encapsulation, and new quality control tests, such as the physical-chemical test, were carried out in a compounding pharmacy that has the green seal of quality, the seal of excellence in franchising, and the Sinamm Diplomation. Weighing was computed using an analytical balance.

Both the ginger and the placebo were prepared by a private laboratory, certified by the National Health Surveillance Agency (Agência Nacional de Vigilância Sanitária, ANVISA), in accordance with the national regulations for drug preparation. During and after preparation, the researchers had the assistance of pharmacists associated with the Federal University of Piauí.

All the participants were encouraged to continue taking routine medications, according to the medical recommendations, and to remain with the same eating and exercise habits during the intervention. They were also informed of the risks and benefits of the study, and the participants were aware that they could leave the research at any time and for any reason, without prejudice to the health treatment offered by the PHCU. All this information was extended to the participants’ family and/or caregivers.

After recruitment, assessment of the eligibility criteria, and people accepting to be part of the study, the researchers scheduled a date to start data collection. Collection was divided into two stages. In the first stage, the participants received instructions about the study and provided socio-economic, clinical, and laboratory data. For data collection, a questionnaire was used containing socioeconomic variables (gender, skin color, schooling level, years of study, marital status, occupation, and income), clinical variables (mean values of systolic and diastolic blood pressure, time of diagnosis with T2DM, episodes of hypoglycemia and hyperglycemia in the last 30 days before the start of the intervention, frequency of follow-up in the PHCU, and physical exercise), and laboratory variables [fasting blood sugar (FBS), HbA1c, low density cholesterol (LDL), high-density cholesterol (HDL), total cholesterol (TC), triglycerides (TG), and HOMA-IR index]. To measure adherence, the Morisky and Green expanded test and the Batalla-Martinez test were used. As it was self-reported, the diverse information provided during data collection in the PHCU could have a response bias.

Blood Pressure (BP) was measured three times and, after that, the mean value was established. The reference levels used are in accordance with the VII Brazilian Guideline for Hypertension^(^ 18 ^)^. The laboratory data corresponded to the levels established by the Brazilian Society of Diabetes^(^ 15 ^)^, and by the V Brazilian Guideline on Dyslipidemias and Prevention of Atherosclerosis^(^ 19 ^)^. Blood samples (10 mL) were taken after 10 to 12 hours of fasting. The samples were centrifuged at room temperature, at 3,000 rpm for 10 minutes, to separate the serum from the blood cells. FBS, TG, TC, LDL, HDL, and HbA1c were determined by the enzymatic colorimetric method with commercially available kits (Pars Azmun Co., Tehran, Iran) in an automated analyzer (Abbott, Alcyon 300 model, Abbott Park, IL, USA). In turn, for HOMA-IR, calculated by multiplying blood glucose by insulin (µUI/mL), both during fasting, and dividing by 22.5, the established cut-off point was 2.5^(^ 20 ^-^ 21 ^)^.

The venipuncture, manipulation, and analysis of the biological samples were made by trained professionals, and the analysis was conducted in a clinical analysis laboratory with the CONTROLLAB quality seal, intermediated by the Brazilian Society of Clinical Pathology and Laboratory Medicine, and the National Quality Control Program quality seal.

For absent participants, a telephone call or home visit was made to recruit them and schedule new dates for participation. During the follow-up period, the participants received a telephone call per month to remind them of the importance of medication adherence, as well as for the lead researcher to record adverse events. Three months after the delivery of the first bottle containing ginger or placebo capsules, the participants once more provided information regarding the clinical and laboratory variables.

**Figure 1:**
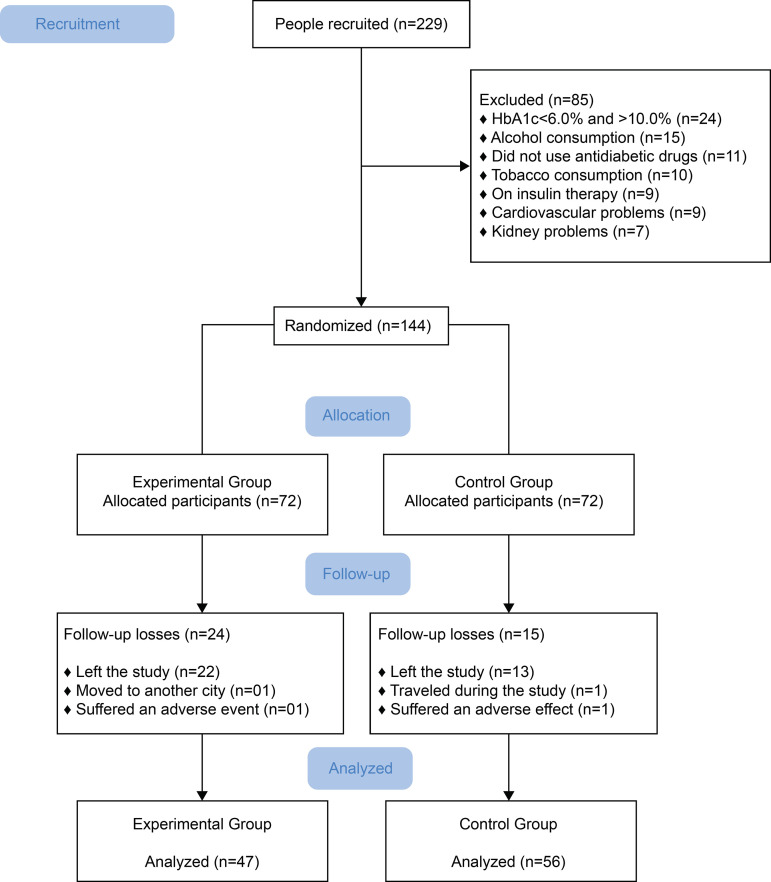
Flowchart of the participants included in the study. Picos, PI, Brazil, 2018

In this research, 142 participants were randomized, 72 in the EG and 72 in the CG. However, only 103 individuals completed the entire treatment (Figure 1). The reason for the losses was linked to discontinuity, outliers or adherence to intervention below 80%. The adverse events presented were the following: diarrhea (n=1) and gastrointestinal discomfort (n=1).

Data was analyzed by protocol. For the continuous variables, data was presented as mean and standard deviation or as median, minimum, and maximum. In the categorical variables, data was exposed in frequency and prevalence rate, in order to investigate associations between risk factors and disease. The Mann-Whitney U test was used to analyze the characteristics of the groups. To verify the behavior of the numerical variables, at both times, the Wilcoxon test was used. A significance level of 5% was adopted. In these cases, the normality and homoscedasticity of the variables were observed using the Kolmogorov-Sminorv and Levene tests, respectively. Based on this, the choice of each test considered the characteristics of the variables.

For the categorical variables, Pearson’s chi-square test and Fisher’s exact test were used to investigate the association between the variables. Data was entered twice, by different members of the research group. Statistical analyses were performed using the Statistical Package for the Social Sciences (SPSS) statistical program, version 22.0 (USA) and the R 3.3.1 software.

In the case of the variances with statistical significance, a multivariate analysis (linear or multinomial regression, depending on each case) was performed to determine the casual relationship between predictive factors and biochemical outcomes under study.

The study was conducted in accordance with Resolutions No. 466/12 and 510/2016 of the National Health Council, approved in the Research Ethics Committee of the State University of Piauí under No. 2,248,450 (CAAE 71423617.3.0000.5209), and recorded with the Brazilian Network of Clinical Trials (RBR-2rt2wy/TRIAL: U111-1202-1650).

## Results

Most of the study participants had a mean age of 58.64 years old (SD±11.11), were female (69.9%), brown-skinned (54.4%), retired or unemployed (61.2%), married/in a stable union (60.2%), with a mean of nine years of study (43.7%), and a monthly income equal to or less than the minimum wage (52.4%), considering the reference value in 2018 (R$ 954.00/USD 243.00).

It was also possible to identify that most of the participants had two to five years of diagnosis (40.8%), were followed up by physicians and nurses on a quarterly basis (43.7%), and most of their follow-up tests occurred approximately twice a year.

With the exception of diastolic blood pressure, the groups were homogeneous with respect to the variables: duration of diabetes, presence of arterial hypertension, follow-up in the PHCUs, physical exercise, hypoglycemia, hyperglycemia, and systolic blood pressure before the intervention (Table 1).

**Table 1:** Characterization of the participants with DM2 according to the clinical variables. Picos, PI, Brazil, 2018

Variables	Total (n=103)	Control Group (n=56)	Experimental Group (n=47)	*p*
Time with diabetes				0.670*
2-5 years	42 (40.8%)	22 (39.3%)	20 (42.6%)	
5-10 years	33 (32.0%)	20 (35.7%)	13 (27.7%)	
> 10 years	28 (27.2%)	14 (25.0%)	14 (29.8%)	
Hypertension				0.274*
Yes	62 (60.2%)	31 (55.4%)	31 (66%)	
No	41 (39.8%)	25 (44.6%)	16 (34%)	
Follow-up in the PHCU				0.558*
Monthly	18 (17.5%)	09 (16.1%)	09 (19.1%)	
Quarterly	45 (43.7%)	28 (50.0%)	17 (36.2%)	
Semiannual	26 (25.2%)	12 (21.4%)	14 (29.8%)	
Others	14 (13.6%)	07 (12.5%)	07 (14.9%)	
Physical Activity				
Before the Intervention	38 (35.9%)	21 (37.5%)	17 (36.2%)	0.889*
After the Intervention	35 (34.0%)	19 (33.9%)	16 (34.0%)	0.990*
Hypoglycemia in the last 30 days				0.623*
Yes	04 (3.9%)	03 (5.4%)	01 (2.1%)	
No	99 (96.1%)	53 (94.6%)	46 (97.9%)	
Hyperglycemia in the last 30 days				0.081^†^
Yes	13 (12.6%)	04 (7.1%)	09 (19.1%)	
No	90 (87.4%)	52 (92.9%)	38 (80.9%)	
Systolic blood pressure		0.527^‡^	0.870^‡^	
Before the Intervention	128.27±17.96	125.02±15.93	131.53±19.99	0.146^§^
After the Intervention	128.04±16.52	125.32±14.36	130.77±18.69	0.147^§^
Diastolic blood pressure		0.955^‡^	0.066^‡^	
Before the Intervention	78.78±9.18	77.09±8.90	80.47±9.47	0.039^§^
After the Intervention	76.59±12.10	76.34±9.44	76.85±14.77	0.937^§^

* Chi-square test;

† Fisher's exact test;

‡ Wilcoxon's test;

§ Mann-Whitney's test. 95% confidence interval

The experimental (p=0.196) and control (p=0.171) groups showed no statistically significant differences regarding adherence to the clinical protocol according to the Morisky and Green expanded test (Table 2).

**Table 2:** Adherence to the pharmacological treatment in the participants of both groups. Picos, PI, Brazil, 2018

Variables	Total (n=103)	Control Group (n=56)	Experimental Group (n=47)	*p*
Morisky and Green Expanded				
High adherence	92 (89.3%)	48 (85.7%)	44 (93.6%)	0.196*
Medium adherence	11 (10.7%)	08 (14.3%)	03 (06.4%)	
Batalla-Martinez				
Adherent	55 (53.4%)	27 (48.2%)	28 (59.6%)	0.171^†^
Non-adherent	48 (45.6%)	29 (51.8%)	19 (40.4%)	

* Fisher's exact test;

† Pearson's chi-square test; 95% confidence interval

In the intra- and inter-group comparison of glycemic and lipid mean values, the participants of the EG showed better results in reducing the levels of FBS, HbA1c, and TC compared to the CG; however, only FBS had a significant attenuation in the intragroup analysis. There was also a better outcome in the HDL levels of the EG when compared to the CG, although not statistically significant. The LDL values showed a better result in those who received a placebo. In the intergroup analysis, however, no outcome variable was significant. It is highlighted that the studied groups were homogeneous regarding the outcome variables (Table 3).

**Table 3:** Intra- and inter-group comparison of participants' glycemic and lipid mean values. Picos, PI, Brazil, 2018

Variables	Before	After	Difference	*p*
Fasting blood sugar				
Control Group	185.23 ± 74.16	175.98 ± 72.57	-9.25 ± 48.44	0.041^†^
Experimental Group	203.60 ± 88.24	174.05 ± 64.10	-29.55 ± 53.76	0.001^†^
p-value	0.297*	0.931*	0.130*	
HbA1c				
Control Group	8.36 ± 1.89	8.29 ± 1.86	-0.06 ± 1.05	0.361^†^
Experimental Group	8.40 ± 1.96	8.14 ± 1.81	-0.26 ± 1.05	0.144^†^
p-value	0.853*	0.721*	0.765*	
Total cholesterol				
Control Group	210.16 ± 57.53	202.07 ± 63.58	-8.09 ± 75.32	0.040^†^
Experimental Group	195.36 ± 46.59	183.74 ± 43.4	-11.62 ± 30.55	0.010^†^
p-value	0.160^†^	0.073*	0.884*	
HDL^‡^				
Control Group	50.00 ± 11.01	48.88 ± 10.1	-1.13 ± 11.87	0.516^†^
Experimental Group	48.04 ± 11.03	50.30 ± 10.9	2.26 ± 9.03	0.098^†^
p-value	0.372^†^	0.112*	0.684*	
LDL^§^				
Control Group	126.79 ± 42.81	114.81 ± 57.06	-11.97 ± 66.15	0.001^†^
Experimental Group	114.21 ± 32.08	106.76 ± 28.43	-7.45 ± 23.12	0.018^†^
p-value	0.100^†^	0.716*	0.120*	
Triglycerides				
Control Group	222.41 ± 130.95	217.71 ± 133.18	-4.70 ± 92.67	0.712^†^
Experimental Group	178.83 ± 94.96	174.19 ± 94.44	-4.64 ± 62.16	0.958^†^
p-value	0.072*	0.131*	0.776*	
HOMA-IR				
Control Group	3.01 ± 1.91	3.20 ± 2.10	0.19 ± 1.75	0.563^†^
Experimental Group	2.99 ± 2.10	3.21 ± 1.90	0.27 ± 2.27	0.251^†^
p-value	0.770*	0.830*	0.586*	

* Mann-Whitney's test;

† Wilcoxon's test;

‡ HDL = HDL cholesterol;

§ LDL = LDL cholesterol; 95% confidence interval

After adjusting the FBS, TC, HDL and LDL outcomes, in both groups, for confounding variables, we detected important findings. In the CG, the total cholesterol variable seems to be influenced by skin color, SAH, and follow-up in the PHCU, whereas the HDL variable appears to be influenced by skin color, economic class, marital status, and SAH. In the EG, FBS is influenced by the time with T2DM and follow-up in the PHCU, and the LDL variable, by gender. It is highlighted that the residual R^2^ of this model was 53.2% (Table 4).

**Table 4:** Model for adjustment of the fasting blood sugar, total cholesterol, HDL, and LDL outcomes, according to confounding variables. Picos, PI, Brazil, 2018

Control Group	FBS	CT	HDL	LDL	Experimental Group	FBS	CT	HDL	LDL
Constant	0.276	0.022	0.005	0.115	Constant	0.972	0.005	0.049	0.002
Age*	0.617	0.135	0.052	0.315	Age	0.190	0.413	0.711	0.283
Gender^†^	0.578	0.294	0.530	0.342	Gender	0.380	0.110	0.653	0.036^‡^
Skin color^†^	0.136	0.022^‡^	0.015^‡^	0.036	Skin color	0.351	0.760	0.727	0.858
Years of study*	0.392	0.777	0.979	0.961	Years of study	0.298	0.796	0.274	0.515
Schooling^†^	0.467	0.669	0.598	0.777	Schooling	0.357	0.900	0.604	0.304
Employed^†^	0.216	0.503	0.402	0.336	Employed	0.836	0.605	0.628	0.831
Monthly Income*	0.104	0.627	0.605	0.399	Monthly Income	0.453	0.308	0.202	0.140
Economic Class^†^	0.240	0.213	0.037^‡^	0.311	Economic Class	0.115	0.928	0.599	0.600
Marital status	0.999	0.322	0.048^‡^	0.402	Marital status	0.231	0.902	0.581	0.459
Who they live with^†^	0.594	0.691	0.408	0.915	Who they live with	0.213	0.219	0.466	0.089
Time with DM2*	0.096	0.502	0.662	0.499	Time with DM2	0.009^‡^	0.970	0.954	0.584
Arterial Hypertension	0.398	0.013^‡^	0.040^‡^	0.038	Arterial Hypertension	0.066	0.176	0.630	0.191
Monitored in the PHCU^†^	0.211	0.045^‡^	0.115	0.151	Follow-up in the PHCU	0.025^‡^	0.500	0.509	0.475

* Simple lineal regression;

† Multinomial regression;

‡ p < 0.05

## Discussion

The results of this study showed that, in doses of 1.2 g daily for 90 days, ginger was effective in reducing FBS and TC values in people with T2DM compared to a placebo, confirming part of the original hypothesis. Among the CG participants, only the decrease in the LDL values was greater than in those of the EG. It is highlighted that medication adherence remained higher in individuals in the EG, in both tests, although without statistical significance. A number of research studies that investigated the effect of ginger in the treatment of T2DM, in different doses and intervention periods, also showed reduced FBS and TC values^(^ 9 ^,^ 11 ^,^ 22 ^-^ 23 ^)^.

The participants in the EG had a reduction in the FBS levels higher by 20.3 mg/dL than those in the CG. Other investigations that analyzed the effect of ginger on FBS also indicated greater reductions in the groups that received the intervention with the herbal drug compared to placebo groups. Such evidence suggests that ginger has therapeutic potential in the treatment of poorly controlled T2DM^(^ 9 ^-^ 10 ^,^ 22 ^-^ 25 ^)^.

Regarding the levels of HbA1c, the attenuation of the values of this variable was greater in the EG than in the CG, although the results were not statistically significant. Although FBS has a direct relationship with HbA1c and has dropped in the EG, the same does not seem to have happened with the HbA1c levels, which may indicate that ginger does not have a linear effect over a long period of time. However, the literature is not unanimous^(^ 25 ^)^, and different clinical trials have shown that daily consumption of ginger causes a reduction in the HbA1c values when offered in larger doses^(^ 9 ^,^ 13 ^,^ 17 ^,^ 24 ^)^. It is important to note that the level of adherence and the participants’ lifestyle habits may have influenced the outcome of this variable.

Regarding the secondary outcomes, it was noticed that there was an increase in insulin resistance in both groups. This fact can be seen by the increase in the HOMA-IR values, although without statistical significance. At higher doses and intervention periods, researchers have shown that ginger has contributed to lower insulin resistance in people with T2DM, which is favorable to reduce complications related to the disease^(^ 10 ^,^ 13 ^,^ 17 ^,^ 26 ^-^ 27 ^)^.

Particularly for the analysis of lipid parameters, when investigating the effectiveness of ginger in reducing these biomarkers, it was observed that only the TC and LDL values showed a statistically significant decrease. However, LDL showed greater attenuation among those who received placebo (p=0.001).

Based on the adjustment model, it is important to highlight the role of time with diabetes and follow-up in the PHCU as adjuvants to the effect of using ginger on the participants of this research. In turn, in the case of the LDL variable, it is important to highlight the association with the participants’ gender. In Brazil, for example, there is data from comprehensive research that found women to be more vulnerable to higher levels for this marker^(^ 28 ^)^.

A systematic review that investigated the effects of ginger on the lipids of people with diabetes showed that, in addition to TC and LDL, the product was able to reduce TG levels when in doses of 1 to 3 g/day^(^ 23 ^)^. However, in this study, although the TG values have dropped compared to the baseline, data are not statistically significant. It was also analyzed whether ginger supplementation could raise HDL levels in the investigated sample. When increased, HDL helps to protect against the onset of atherosclerotic disease in people with diabetes^(^ 22 ^)^. In the EG, the results showed that HDL increased by 2.26 mg/dL after 90 days of intervention, although without statistical significance.

The assessment of lipid markers in people with diabetes is important given the complications arising from the accumulation of fat, such as obesity, hypertriglyceridemia, metabolic syndrome, atherosclerosis, and other cardiovascular diseases. Although only some of the lipid variables investigated in this study have dropped, other clinical trials validate that the supplementary use of ginger is effective in reducing the lipid ranges in people with T2DM^(^ 13 ^)^, even though the results are heterogeneous^(^ 9 ^,^ 29 ^)^. It is noted that, in this study, the homogeneity of the groups regarding the socioeconomic and clinical variables reduced possible biases in differential and confounding information.

Although R^2^ (53.2%) did not attain a very high level in the adjustment model, some predictor variables proved to be statistically significant when associated with the outcome variables. Even so, it is still possible to draw conclusions about the influence of these predictor variables on the studied outcomes.

The limitations of this study were the intervention period and the dose offered. Although most of the existing studies establish 90 days, longer follow-ups could show different outcomes and allow for a better assessment of ginger in the treatment of T2DM. In addition, in Brazil, the maximum permissible dose for daily consumption of ginger is 1.2 g of dry extract, which made it impossible to find conclusions closer to those demonstrated in foreign research studies. It is also not possible to generalize the results of this study, since the sample is limited to people monitored in PHCUs and who live in a city in the inland of the state of Piauí, with a different diet and lifestyle from individuals who live in large urban centers.

It is highlighted that, during the intervention period, only two participants reported some adverse event, one in the EG and the other in the CG. Data from two systematic reviews were unanimous regarding the safety of this product, and they minimize the occurrence of toxicity from the use of ginger^(^ 13 ^,^ 25 ^)^.

From observation and interpretation, it is possible to say that ginger has therapeutic potential to be used in the treatment of T2DM, increasing the chances of normal levels of blood sugar and lipids in people with the disease.

Thus, the most relevant contribution of this research is to make it evident that the use of ginger is viable as an adjuvant herbal drug in the treatment of T2DM, especially because it is an easily accessible and low-cost spice, which can serve as a complementary technology to be offered in the clinical practice of nurses, supporting the work of these professionals, and encouraging equitable, integral, and resolute practices also by the multidisciplinary team that works in Primary Health Care^(^ 30 ^)^. Furthermore, this clinical trial is a pioneering initiative in the country, since it fills not only a knowledge gap, but also elucidates possible limitations that can be adjusted in future research studies.

## Conclusion

This study demonstrated that the daily consumption of 1.2 g of ginger for 90 days decreased the values of FBS, TC, and LDL in people with T2DM. Longer research studies using doses higher than the one presented in this research, considering different metabolic variables, and assessing the cost-effectiveness of ginger, should be explored.
